# A Label-Free Fluorescent Array Sensor Utilizing Liposome Encapsulating Calcein for Discriminating Target Proteins by Principal Component Analysis

**DOI:** 10.3390/s17071630

**Published:** 2017-07-15

**Authors:** Ryota Imamura, Naoki Murata, Toshinori Shimanouchi, Kaoru Yamashita, Masayuki Fukuzawa, Minoru Noda

**Affiliations:** 1 Graduate School of Science and Technology, Kyoto Institute of Technology, Matsugasaki, Sakyo-ku, Kyoto 606-8585, Japan; m5621003@edu.kit.ac.jp (R.I.); m5622050@edu.kit.ac.jp (N.M.); yamashita.kaoru@kit.ac.jp (K.Y.); fukuzawa@kit.ac.jp (M.F.); 2 Graduate School of Environmental and Life Science, Okayama University, 1-1-1 Tsushima-naka, Kita-ku, Okayama 700-8530, Japan; tshima@cc.okayama-u.ac.jp

**Keywords:** biosensor, fluorescence, liposome, protein, array, interaction, cholesterol, principal component analysis (PCA)

## Abstract

A new fluorescent arrayed biosensor has been developed to discriminate species and concentrations of target proteins by using plural different phospholipid liposome species encapsulating fluorescent molecules, utilizing differences in permeation of the fluorescent molecules through the membrane to modulate liposome-target protein interactions. This approach proposes a basically new label-free fluorescent sensor, compared with the common technique of developed fluorescent array sensors with labeling. We have confirmed a high output intensity of fluorescence emission related to characteristics of the fluorescent molecules dependent on their concentrations when they leak from inside the liposomes through the perturbed lipid membrane. After taking an array image of the fluorescence emission from the sensor using a CMOS imager, the output intensities of the fluorescence were analyzed by a principal component analysis (PCA) statistical method. It is found from PCA plots that different protein species with several concentrations were successfully discriminated by using the different lipid membranes with high cumulative contribution ratio. We also confirmed that the accuracy of the discrimination by the array sensor with a single shot is higher than that of a single sensor with multiple shots.

## 1. Introduction

Important biomolecules such as DNA, RNA, proteins and so on are commonly detected by fluorescence techniques based on labeling and staining [1,2,3]. Both the fluorescent antibody and the immunofluorescent technique are major techniques in the field of biochemistry because they offer high detectability, stability and safety. Especially, immunofluorescence methods such as enzyme-linked immunosorbent assays (ELISA) have been used to detect various proteins [4,5,6] with high sensitivity. However, ELISA methods involve quite complicated operations, user proficiency, large device size and so on. Therefore, we have attempted to develop new simplified and label-free fluorescent sensing techniques, utilizing phospholipid membrane liposomes, although several immune assays based on liposomes have been reported so far such as a liposome immunosorbent assay and a liposome immune lysis one [7,8].

We have reported preliminary results on an arrayed biosensor utilizing liposomes to encapsulate fluorescent molecules and the time course analysis of the fluorescence [9]. The phospholipid bilayer of the liposome is used as a model cell membrane sensing biomolecule to detect and discriminate external target biomolecules. It is known that the encapsulated molecules leak from the internal aqueous phase after the interactions [10,11]. The phenomenon has been used to characterize the membrane properties and evaluate the interaction against external biomolecules such as proteins [12,13], peptides [14] and the others [15]. In the operation of the fluorescent liposome biosensor, the leakage of fluorescent molecules increases the fluorescence intensity.

Therefore, it is further required: (1) to achieve a higher and more stable output intensity of fluorescence emission; (2) to investigate more different types of liposome phospholipid to improve the sensitivity and discrimination capability between different target biomolecules, and (3) to analyze the arrayed data statistically to increasing the accuracy of the analyzed results.

In this work, we firstly considered a proper concentration of fluorescent calcein molecule for encapsulation in the liposome, as calcein encapsulation is often used to evaluate membrane permeation [16,17,18]. It is necessary to know the proper calcein concentration in order to emit enough fluorescence when the calcein leaks from the liposome by the liposome-target molecule interaction. Secondly, an important component of cell membrane, cholesterol, was newly incorporated into the liposome membrane because the membrane fluidity changes significantly by incorporation of cholesterol [19,20], which influences on the interaction between the membrane and target molecules. Thirdly, we also evaluated the interaction strengths of different phospholipid liposome species with target molecules to identify the most effective phospholipids. Finally, we proceeded to apply principal component analysis (PCA) for statistical data analyses [21], since most of the microarray techniques need comprehensive analysis because those techniques obtain multi-dimensional data and extract characteristic results. Using PCA, we examined the feasibility and applicability of arrayed sensors with single shots in comparison with single microwell sensors with multiple shots.

## 2. Materials and Methods

### 2.1. Materials

The phospholipids we used were 1,2-dipalmitoyl-*sn*-glycero-3-phosphocholine (DPPC, MW = 734.04), 1,2-distearoyl-*sn*-glycero-3-phosphocholine (DSPC, MW = 790.15). Besides, we used DPPC incorporating cholesterol (MW = 386.65) (DPPC:cholesterol = 66 mol%:33 mol%). DPPC, DSPC, and cholesterol were purchased from Avanti Polar Lipids (Alabaster, AL, USA). Target proteins we used were bovine carbonic anhydrase (CAB, MW = 28,400) and lysozyme (MW = 17,307), which were purchased from Sigma Aldrich (St. Louis, MO, USA). The fluorescent molecule encapsulated in liposomes was calcein (MW = 622.53), which was also purchased from Sigma Aldrich. Silpot184 and Silpot184 CAT (hardener) purchased from Toray Dow Corning (Tokyo, Japan) were used for the fabrication of polydimethylsiloxane (PDMS)-based chips. The gel beads, Sepharose 4B, which are used for filtering out the free calcein during liposome preparation, were purchased from GE Healthcare (Uppsala, Sweden). The filtration buffer, phosphate buffered saline (PBS), were purchased from Thermo Fisher Scientific (Yokohama, Japan). All the chemical reagents were of analytical grade.

### 2.2. Preparation of Liposomes

Three kinds of liposome were prepared using DPPC, DSPC, and DPPC incorporating cholesterol (DPPC:cholesterol = 66 mol%:33 mol%) in the same manner as previously reported [10,11,12,22]. In brief, three kinds of lipid solutions were prepared in chloroform. They were dried in round-bottom flasks by rotary evaporation under reduced pressure. The obtained homogeneous lipid films were kept under vacuum for more than 3 h and then hydrated with 100 mM calcein solution to form multilamellar vesicles. After five cycles of freezing-thawing treatment, the liposome size was adjusted by the extrusion of liposome suspension through a polycarbonate membrane (pore size: 100 nm). Finally, the free calcein was removed by gel permeation chromatography on Sepharose 4B.

### 2.3. Fabricated Photometric System

**Figure 1:**
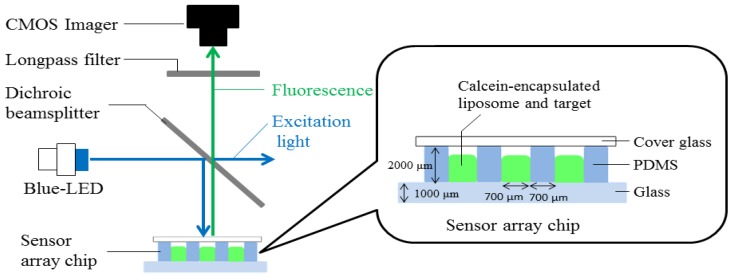
A cross-sectional view of microwells of array sensor with illustration of the fluorescence photometric system. Polydimethylsiloxane (PDMS); Light-Emitting Diode (LED).

**Figure 2:**
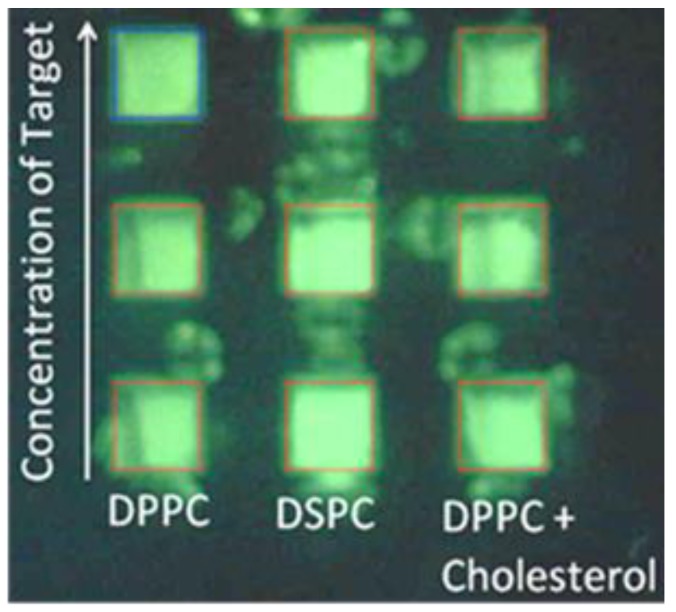
A fluorescent image from the array sensor. Along the horizontal direction, the microwells were filled with different phospholipid liposome species, and along the vertical direction, they were provided with different concentrations of the same kind of target protein. 1,2-dipalmitoyl-sn-glycero-3-phosphocholine (DPPC); 1,2-distearoyl-*sn*-glycero-3-phosphocholine (DSPC).

We constructed a photometric system in order to take fluorescent images of array sensor chips. Figure 1 shows a cross-sectional view of the microwells of the sensor array with an illustration of the fluorescence photometric system. Figure 2 shows a fluorescence image from the array sensor. In the measurement, each microwell was filled with a common liposome suspension (10 mM) encapsulating calcein (100 mM) together with a target protein of different species and concentration from each other, thereafter the array was covered with a transparent cover glass. Calcein excitation light (around 495 nm in wavelength) emitted from a blue Light-Emitting Diode (LED) (λ = 495 nm), is reflected by a dichroic beamsplitter and reaches the arrayed microwells. Owing to liposome-protein interaction, calcein molecules leak from the internal aqueous phase of the liposomes, thereafter the fluorescence of the leaked calcein is emitted from the arrayed microwells, passes through the dichroic beamsplitter and a 502 nm longpass filter, successively, and is finally detected by a CMOS imager (WAT-01U2, Watec. Co., Ltd., Tsuruoka, Japan). We evaluated the liposome-protein interaction by investigating the change of intensity of fluorescence emissions using our developed software for analysis [23].

## 3. Results and Discussions

### 3.1. Optimization of Fluorescence of Calcein in Arrayed Microwells

It is known that fluorescence intensity depends on the fluorescent molecule concentration, especially for calcein [24,25]. In our case, the calcein molecules are released depending on how much the liposome membrane permits their permeation. Thus, it is important to prepare calcein with a proper concentration encapsulated in the liposome for increasing and optimizing the fluorescence intensity. To investigate the dependence on the concentration, we prepared calcein solutions diluted by phosphate buffered saline (PBS) with concentration of 0.001, 0.01, 0.1, 0.5, 1, 5, 10, 50, and 100 mM, respectively. After the calcein solutions were introduced into the arrayed microwells, fluorescence measurements were carried out using a fluorescent microscope (BX51, OLYMPUS, Tokyo, Japan) and a halogen lump (U-HGLGPS, OLYMPUS) as an excitation light source filtered at 460–495 nm.

**Figure 3:**
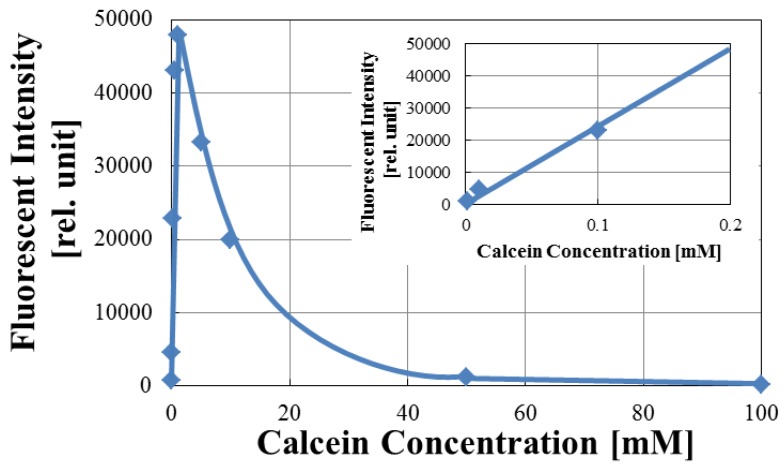
Fluorescent intensity dependence on calcein concentration.

Figure 3 shows the fluorescent intensity measured in the arrayed microwells as a function of calcein concentration. The fluorescent intensity of calcein solution increases rapidly and linearly in low concentration range (as seen in the inset) and is maximum at around 2 mM. For concentrations higher than 2 mM, the fluorescent intensity decreases with increase in concentration. In particular, the fluorescent intensity became almost zero when the concentration increased to 100 mM. This phenomenon where calcein is quenched at high concentrations is called ‘self-quenching’ [24,25,26]. Accordingly, we set the calcein concentration encapsulated in liposome as high as 100 mM. In this experiment, based on our our previous results the calcein molecules are estimated to leak by less than a few percent, so the fluorescence intensity of the leaked calcein is much higher than that of the liposomed encapsulating 100 mM calcein.

### 3.2. Effect of Cholesterol on Liposome-Protein Interaction

We measured the liposome-protein interaction by the developed photometric system, and investigated the effect of incorporating cholesterol on the interaction. The suspension of liposome encapsulating 100 mM calcein added with target protein (CAB or lysozyme) was introduced into the arrayed microwell, thereafter the surface of the array chip was sealed by a transparent cover glass. The fluorescence image from the array is shown again in Figure 2. Along the horizontal direction, the microwells were filled with different phospholipid liposome species, and along the vertical direction, they contained different concentrations of the same kind of target protein. Differences in fluorescent intensity among the microwells can be observed dependending on the phospholipid species and concentration of target protein. We investigated the time course of the fluorescence intensity of leaked calcein caused by the interaction between liposomes incorporated with cholesterol and target proteins for 60 min by the photometric system. Moreover, we calculated relative change of fluorescence intensity against the initial intensity (at the start of the measurement). The relative change of fluorescence intensity, defined as RF, is calculated as shown in Equation (1):

(1) $$\mathrm{RF}=\;\frac{\Delta I}{I_{0}}=\;\frac{I-\;I_{0}}{I_{0}}$$

Here *I* is fluorescence intensity after 60 min from the start, and *I*_0_ is the initial fluorescence intensity (at 0 min). Table 1 lists averaged RF’s of liposome suspensions of DPPC and DPPC/cholesterol added with 100 μM and 300 μM CAB after 60 min from the start. Regardless of the different liposomes used, RF increases with the increase in CAB concentration. This means that the amount of leaked calcein molecules increases with the concentration of target protein. Also, the RF of DPPC/cholesterol is higher than that of DPPC for the same concentration of CAB. We consider reasonable that incorporating cholesterol into the phospholipid membrane improves the fluidity of liposome. This would lead the observed improvement in sensitivity.

**Figure 4:**
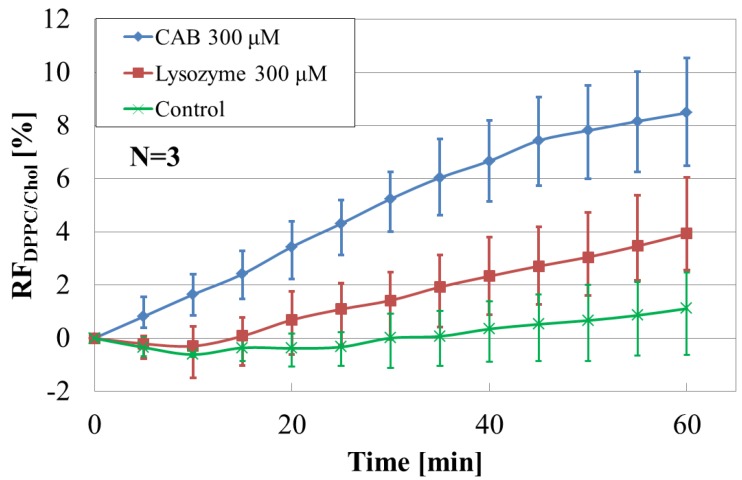
Time course of RF’s of DPPC/cholesterol added with different target proteins (300 μM) for 60 min, including the results of control sample without those proteins.

Next, Figure 4 shows the time course of RF’s of DPPC/cholesterol added with different target proteins at the same concentration (300 μM), also including the result of a control sample without the presence of those proteins. As mentioned in 3.1, it is noted for the control sample that the calcein molecules are estimated to leak by less than about 2%. From the figure, it is found that RF increased with time and the liposome-CAB interaction is larger than the liposome-lysozyme interaction. We consider that the difference in the behavior of liposome-protein interaction originates from that in the molecular structures of the proteins such as different amount of disulfide (S-S) bonds [27,28,29]. It is reasonable that the number of S-S bonds influences the intensity of interactions. The lysozyme molecule has four disulfide bonds, thus it has high structural stability and exposing its hydrophobic groups is difficult. However, CAB molecules have no disulfide bond, so the hydrophobic groups of CAB are easily exposed. Therefore, the hydrophobic interaction between the liposome and CAB is stronger than that between the liposome and lysozyme.

### 3.3. Effect of Phospholipid Species on Liposome-Protein Interaction

**Figure 5:**
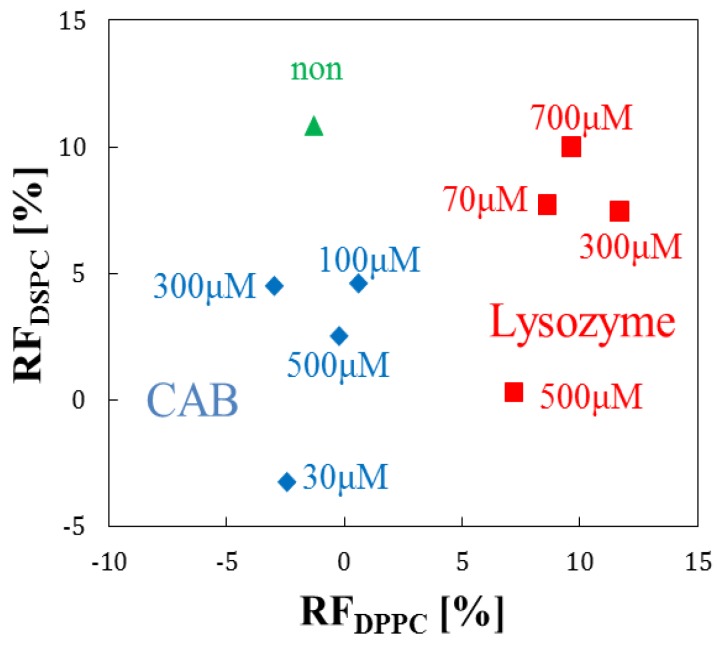
Scatter plot of averaged RF’s of DSPC and DPPC after 60 min from the initial addition of target proteins.

We investigated the time course of RF’s of liposome suspensions of DPPC, DPPC/cholesterol, and DSPC added with target proteins for 60 min. In the measurement, we selected 30, 100, 300, and 500 μM as the concentration of CAB, and also 70, 300, 500, and 700 μM as the concentration of lysozyme, respectively. Figure 5 plots the averaged RF’s of DSPC vs. that of DPPC measured after 60 min from the initial addition of the target proteins. From Figure 5, it is clear that the RF plots of each different target protein are well separated. Moreover, the plots are also separated and discriminated between the different concentrations. The difference in RF between different phospholipid liposomes is reasonably due to the structure of phospholipid: DPPC and DSPC are composed of saturated fatty acids, which carbon numbers are 16 and 18, respectively. Since the structural stability of lipid molecules with long saturated fatty acid chains is high and the corresponding fluidity becomes low [30], the interaction strength of DPPC is larger than that of DSPC. It is noted from Figure 5 that the RF values of DPPC are relatively higher than those of DSPC, suggesting that the difference of liposome-target protein interaction was caused by that between the species of phospholipid. On the other hand, in Figure 5, it should be noted that the highest RF corresponds to a median concentration, not the highest one. One of the reasons is considered to be that chronological behavior of the calcein leakage would not be always a monotonous increase with time and not proportional to the concentration. It is possible that the behavior would show a specific state of interaction between each different phospholipid and target protein. Another reason may be also due to the fluctuations between different measurements with the same phospholipid species and target protein. Therefore, the above result suggests that it is questionable whether we can obtain a reliable correlation from only simple RF plots. We think that an effective approach is to improve the statistical procedure.

The PC group shows neutrality and no electrostatic interaction ability, therefore it is inert to many proteins, suggesting low sensitivity, although we have used the neutral PC membrane because of the simplicity of its experimental treatment and application in sensor devices. As one of effective method, we have proposed the addition of cholesterol into the PC membrane, mentioned above. The exploration and development of different species and surface modification of phospholipid membranes are still preliminary at the present stage.

Here we should consider an osmotic effect due to the high levels of proteins found in the extravesicular environment. Firstly, the possibility of osmotic effect due to the addition of high levels of proteins cannot be ruled out from all our experiments. Osmotic effects depend on the protein concentration according to the van’t Hoff equation: П = *C*_pr_*RT* where П, osmotic pressure; *C*_pr_, protein concentration; *R*, gas constant; *T*, absolute temperature. If the calcein leakage observed in this work was simply induced by the osmotic effect due to the addition of proteins, the RF value obtained for CAB (300 µM) should be same as that for lysozyme (300 µM). Meanwhile, the time course of RF value for CAB showed was different from that seen for lysozyme, as shown in Figure 4. Thus, the difference in RF value between CAB and lysozyme suggests a difference in the corresponding protein-liposome interactions.

We have previously investigated the protein-liposome interaction by other techniques. We immobilized PC-liposome entrapping calcein into gel beds. CAB and lysozyme were loaded into the PC-liposome-immobilized gel bed. The elution time of CAB was definitely different from lysozyme under strong chemical conditions (such as pHs or denaturants) although small differences in elution time between both was observed under mild conditions (neutral pHs) [31]. Under such mild conditions, we detected the protein-liposome interaction using dielectric dispersion analysis [32]. Protein-liposome interaction (protein 10 µM, lipid 10 mM) could be detected by the reduction of headgroup mobility of PC in the liposomal interface. The dielectric dispersion analysis made it possible to detect the interaction under mild conditions, although this technique requires experimental conditions that include high lipid concentrations and low ion strength conditions. Consequently, we considered that the protein-liposome interaction, that might involve the osmotic effect to a certain extent, could be detected even in the case of high levels of proteins (300 µM).

### 3.4. Evaluation by Principal Component Analysis

Next, we tried to analyze the obtained data by principal component analysis (PCA). PCA is a dimension reduction approach that has widely used to visualize high dimensional data in metabolomics [33,34]. PCA is a statistical procedure that transforms the data with many variables into a few composition variables without correlation with each other. We select several principal components from the greatest dispersions and analyze the components. The aim of PCA is grouping those correlated variables and replacing the original descriptors by new set called principal components [35]. The principal component scores are described as in Equation (2), where *ω_ij_* is weighted proportion with the condition of Equation (3) [10,11]:

(2) $$PC_{i}=\omega_{i1}RF_{DPPC}+\;\omega_{i2}RF_{DSPC}+\;\omega_{i3}RF_{DPPC/Chol.}$$

(3) $$\omega_{i1}{}^{2}+\;\omega_{i2}{}^{2}+\;\omega_{i3}{}^{2}=1$$

**Figure 6:**
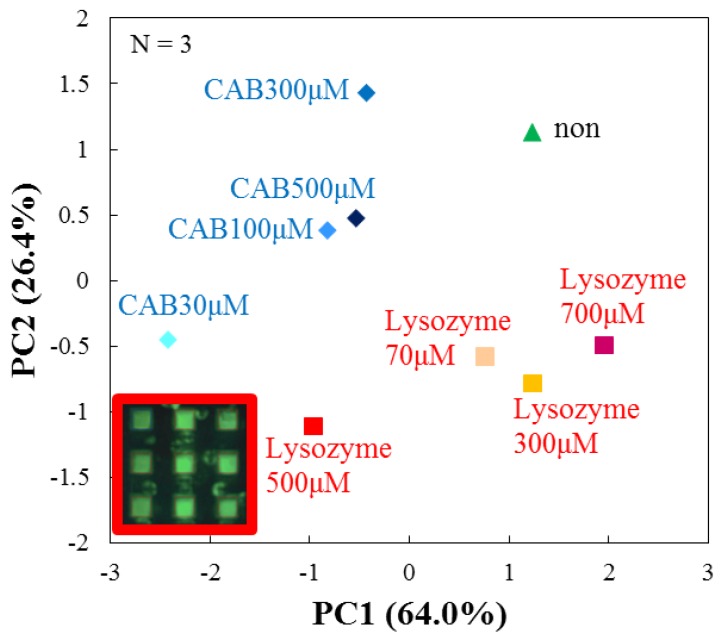
PCA score plot (N = 3) from averaged RF’s of DPPC, DSPC and DPPC/cholesterol after 60 min from the initial addition of target proteins by a single-shot of arrayed microwells.

We used a correlation matrix to analyze the measured RF by PCA, thereby we calculated principal component score and finally we created a PCA score plot. Figure 6 shows a PCA score plot, where averaged results (N = 3) are plotted for each protein as a parameter of concentration. It is found that the two target proteins with different concentrations are clearly discriminated. As cumulative contribution ratios larger than 90% are obtained for both PC1 and PC2, we consider that this analyzed result is sufficient to discriminate between the species and concentrations of target proteins.

### 3.5. Performance Analysis of the Array Sensor

**Figure 7:**
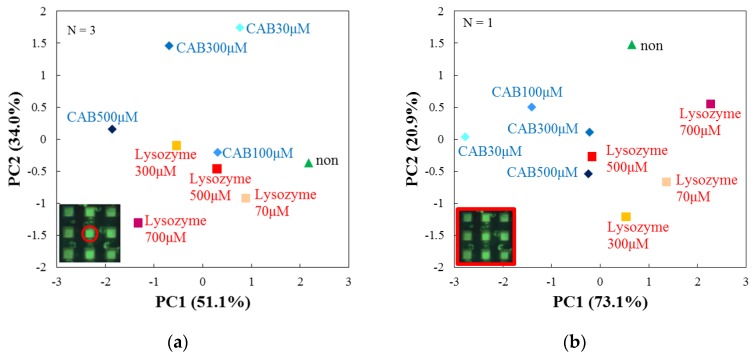
(**a**) PCA score plot (N = 3) of averaged RF’s for multi-shots of single microwell; (**b**) PCA score plot (N = 1) of RF’s for single-shot of arrayed microwells. The RF’s are for DPPC, DSPC and DPPC/Cholesterol after 60 min from the initial addition of target proteins.

Moreover, we examined the performance of arrayed microwells by comparing them with a single microwell. We measured the dispersion across multiple single microwell shots and that across a single-shot of arrayed microwells. Multiple single microwell shots were carried out by sequentially measuring targets in the central microwell of the array chip, focusing on the dispersion that is dependent on different measurements. Single-shots of arrayed microwells were done by simultaneously measuring 9 (=3 × 3) targets in all the microwells of the array, focusing on the dispersion that was dependent on the spatial position of each microwell. Then we compared the dispersions obtained by the two methods. Firstly, we compared the results of the two different measurements analyzed by PCA. Figure 7a shows the result of multiple shots of a single microwell (N = 3). Figure 6 shows again the result of single-shots of arrayed microwells (N = 3). In the case of multiple single microwell shots (Figure 7a), it cannot discriminate the species of target proteins along PC1, because CAB 100 μM cannot be separated from lysozyme 500 μM. The discrimination of the species along PC2 is not good for CAB 100 μM, lysozyme 300 μM, lysozyme 500 μM and no protein. Thus, it is clear that the dispersion dependence on different measurements with single microwells is considerably high. On the other hand, in the case of a single-shot of arrayed microwells in Figure 6, the species are more clearly discriminated along PC1 than the case in Figure 7. Therefore, the results suggest that the dispersion of single-shot arrayed microwells is larger than that of multiple shots of a single microwell in terms of PCA.

Next, we considered the number of measurements for the single-shot of arrayed microwells. Figure 6 and Figure 7b show the results of single-shot of arrayed microwells with N = 3 and 1. For the case of N = 1 (Figure 7b), the protein discrimination is unclear not only along PC1 but also PC2 because some points gather in the center area. However, for N = 3 (Figure 6), the discrimination of target proteins is clearer than that for N = 1, as the plots for CAB maintain a sufficient distance from those for lysozyme. Accordingly, it becomes possible to discriminate the target species and also their concentrations by a single-shot of the array sensor because RFs are well averaged as the number of measurements increases from N = 1 to 3. We again note that the discrimination of protein is more feasible by the arrayed microwells than the single microwell.

At the present stage, we have only detected and discriminated high concentrations of target proteins, much different from the detection level possible with ELISA. The sensitivity and discriminability (specific interaction strength) of phospholipids against target molecules is intrinsic and the most important among all the components of this sensor system. Therefore, the work on exploration and development of specific phospholipid and/or surface modification with functional biomolecules such as specific antibodies, sugar chains and so on should be continued. Also, it is inevitably necessary to increase the number of different targets to prove the specificity of this sensor, as we have discriminated only two types of proteins.

## 4. Conclusions

A fluorescent array sensor utilizing different phospholipid liposomes encapsulating calcein molecules was fabricated to discriminate target proteins and their concentrations. The fluorescence of calcein leaked from inside the liposomes as a result of liposome-protein interactions was detected by a developed photometric system. We examined the proper concentration of calcein encapsulated in the liposome by measuring the fluorescence intensity of calcein as a function of its concentration. Owing to the individual liposome-target protein interactions, the fluorescence intensity time courses obtained from different phospholipid liposomes became different.

We confirmed that: (1) incorporating cholesterol into the phospholipid membrane is an effective way to improve the fluidity of the membrane and the resultant fluorescence, and (2) the fluorescence behaviours of different liposome phospholipids are different. From the measured RF’s, PCA successfully discriminated the species and concentration of target proteins (CAB and lysozyme). Also, the protein discrimination by the arrayed sensor with a single-shot was better than that with multiple shots of a single microwell. At the present stage the system capability allows one to approximate the order of concentration of samples, therefore, we hope the next step is to figure out the concentration of unknown tested samples. Finally, we believe that the developed label-free liposome fluorescent arrayed sensor is effective to discriminate different target proteins with different concentrations.

## Figures and Tables

**Table 1:** Averaged RF’s of liposome suspension of DPPC and DPPC/Cholesterol added with 100 μM and 300 μM CAB after 60 min from the start.

Target Protein (Conc.)	RF(%) for DPPC	RF(%) for DPPC + Cholesterol
CAB (100 µM)	−1 ± 2	−1 ± 2
CAB (300 µM)	2 ± 1	8 ± 2

1,2-dipalmitoyl-sn-glycero-3-phosphocholine (DPPC); bovine carbonic anhydrase (CAB).
